# Difference-Spectrum Completion of Multi-Weight Optical Orthogonal Codes

**DOI:** 10.3390/e28101106

**Published:** 2026-10-10

**Authors:** Jin Uk Park, Seo-Eun Park, Dongsup Jin, Jin-Ho Chung

**Affiliations:** 1 School of ICT Convergence, University of Ulsan, Ulsan 44610, Republic of Korea; 2 Department of Electrical, Electronic and Computer Engineering, University of Ulsan, Ulsan 44610, Republic of Korea; dsjin@ulsan.ac.kr

**Keywords:** optical orthogonal code, cyclic difference packing, Chinese remainder theorem, optical code-division multiple access

## Abstract

Variable-weight (also called multi-weight) optical orthogonal codes (VW-OOCs) with maximum correlation one admit a cyclic difference-packing formulation. We characterize the exact difference spectrum of an existing Chinese-remainder graph construction: each mixed-coordinate difference determines a unique candidate base pair and graph index. A pairwise balanced design covers the mixed region exactly, enabling independent completion on the two coordinate axes. For weights {3,4}, punctured finite geometries give families that are optimal for their prescribed profiles. For every prime p≡1(mod12), the length-12p family has $\left(n_{3},n_{4}\right)=\left(\left(p+1\right)/2,3\left(p-1\right)/4\right)$, profile (2(p+1),3(p−1))/(5p−1), and deficiency 5. For every prime p≡1(mod30), the length-15p family has $\left(n_{3},n_{4}\right)=\left(\left(p-1\right)/2,p\right)$, profile (p−1,2p)/(3p−1), and deficiency 2. Both deficiencies are the minima permitted by congruence. This study’s contribution is the exact-spectrum analysis and its uniform completion mechanism; the lengths and weight set are known, and the second completed parameter tuple follows by filling a previously known regular packing. Explicit examples are verified by exact enumeration.

## 1. Introduction

Optical orthogonal codes (OOCs) are sparse binary sequences designed to have small periodic auto-correlation and cross-correlation values. They were introduced as signature sequences for asynchronous optical code-division multiple access, where the correlation constraints control false synchronization and multi-user interference [1,2]; classical bounds and optimal constructions were subsequently developed in [3]. We use “multi-weight” in the title as a synonym for “variable-weight”; throughout the paper we use the standard abbreviation VW-OOC. Constant-weight OOCs assign the same number of pulses to every user, whereas variable-weight OOCs (VW-OOCs), introduced by Yang [4], permit several Hamming weights in one family and thereby support multiple quality-of-service classes. Their design is naturally connected with cyclic packings and difference families [5,6,7,8,9].

For maximum correlation one, the difference formulation is especially effective. A codeword support contributes its ordered internal differences, and an OOC is obtained precisely when no nonzero difference is repeated across the family. The correlation problem can therefore be expressed in terms of an index-one packing on a cyclic group. It also gives a direct resource count: a block of size *w* uses w(w−1) nonzero differences. Refined Johnson-type bounds combine this difference budget with the prescribed proportion of each weight [6].

Many optimal VW-OOCs with weights {3,4} are already known. Jiang, Wu, and Lee constructed fixed-profile families for complete congruence classes of lengths [10]. One profile is R=(1/3,2/3), and the other is R=(2/3,1/3). General and perfect-difference-family constructions provide related families under these and other profiles [8,11], while Qin and Zhao obtained regular length-12q and length-15q families for primes q=6st+1 under further profiles [12]. Consequently, neither the length forms 12p and 15p nor the weight set {3,4} is new by itself. The relevant comparison must use the complete tuple consisting of the length, exact weight profile, block counts, and difference deficiency; this comparison is made explicitly in Section 3.3.

Pak et al. [13] introduced a construction based on the Chinese remainder theorem (CRT) and a primitive element of a prime field. When p−1=kL, a subset $P\subseteq Z_{k}$ generates graph blocks

(1) $$\left\{(d,\alpha^{dL+i}):d\in P\right\}\subseteq Z_{k}\times F_{p},\;0\leq i<L,$$

where α is primitive in $F_{p}$. Pairwise intersections of the base subsets are restricted to at most one point, which is sufficient to establish maximum correlation one. The published examples emphasize the selection of base subsets for particular weight profiles, but the construction also contains a more rigid difference structure that was not made explicit.

The present paper determines this structure and uses it to complete the code family systematically. For every mixed-coordinate difference, we identify the unique candidate ordered pair in the base design and hence obtain its exact multiplicity. If the base pair packing has *ℓ* uncovered pairs, the graph family has exactly

(2) p+k−2+2Lℓ

uncovered nonzero differences. In particular, a pairwise balanced design covers the entire mixed-coordinate region, leaving only the two coordinate axes. Independent cyclic packings on these axes can then be added without producing repeated differences. A profile-optimality criterion based on the remaining difference deficiency is developed, and punctured PG(2,3) and AG(2,4) designs yield two infinite classes with weights 3 and 4. Besides attaining the refined bound for their prescribed profiles, both classes use the largest number of differences allowed by the congruence condition modulo six. Our contribution is the exact multiplicity characterization and the resulting uniform route from a base pair packing to an axis-completed code. Theorem 3 records both exact profiles and block counts together with profile-constrained optimality and minimum deficiency. These properties do not imply that the tuples are inaccessible to earlier filling constructions; in particular, the length-15p tuple also follows from a regular packing of Jiang, Wu, and Lee, as explained in Section 3.3.

The rest of the paper is organized into three sections: Section 2 presents the difference-spectrum analysis and the coordinate-axis completion theorem. Section 3 develops the finite-geometric constructions, proves optimality, and gives explicit verified examples. Section 4 concludes the paper.

## 2. Difference-Spectrum Framework and Completion

### 2.1. Difference Packings and Profile-Constrained Optimality

Let *N* be a positive integer and let $B\subseteq Z_{N}$. The binary sequence with support *B* has periodic auto-correlation

(3) $$\lambda_{B}\left(\tau \right)=\left|\left(B+\tau \right)\cap B\right|,\;\tau \in Z_{N},$$

and two supports $B,C\subseteq Z_{N}$ have periodic cross-correlation

(4) $$\lambda_{B,C}\left(\tau \right)=\left|\left(B+\tau \right)\cap C\right|.$$

**Definition** **1** (Variable-weight optical orthogonal code)**.** *Let $W=\{w_{1},\ldots ,w_{s}\}$ consist of distinct integers at least two, and let $R=(r_{1},\ldots ,r_{s})$ have positive entries summing to one. An (N,W,1,R) VW-OOC is a family of binary sequences of length N, represented by distinct supports $B_{1},\ldots ,B_{M}\subseteq Z_{N}$, with exactly $r_{j}M$ supports of size $w_{j}$, such that $\lambda_{B_{i}}\left(\tau \right)\leq 1$ for every τ≠0, and $\lambda_{B_{i},B_{h}}\left(\tau \right)\leq 1$ for every i≠h and every $\tau \in Z_{N}$.*

For a block *B*, define its multiset of ordered internal differences by

(5) ΔB=[x−y:x,y∈B,x≠y].

For family B of blocks, put

(6) $$\Delta B=\underset{B\in B}{⊎}\Delta B,\;D\left(B\right)=\mathrm{supp}\left(\Delta B\right).$$

where ⊎ denotes the multiset union, so that multiplicities are retained; equivalently, the multiplicity of each difference in ΔB is the sum of its multiplicities over all B∈B. Thus, D(B) is the set of nonzero differences used by the family.

**Definition** **2.** *Family B of subsets of $Z_{N}$ of size at least two is a *cyclic difference packing of index one* if every nonzero element of $Z_{N}$ has multiplicity at most one in ΔB. Its *difference deficiency* is*

(7) ε(B)=N−1−|D(B)|.

The following standard equivalence is the difference-packing formulation used in [2,6]; its short proof is included for the conventions used here.

**Proposition** **1.** *Let $B=\{B_{1},\ldots ,B_{M}\}$ be a family of subsets of $Z_{N}$ with a size of at least two. The corresponding binary sequences form an OOC with a maximum correlation of at most one if and only if B is a cyclic difference packing of index one.*

**Proof.**  For one block, $|\left(B_{i}+\tau \right)\cap B_{i}|$ equals the multiplicity of τ in $\Delta B_{i}$ with the convention x−y in (5). Hence, a repeated internal difference is equivalent to an out-of-phase auto-correlation of at least two. For distinct blocks, suppose that $\left(B_{i}+\tau \right)\cap B_{j}$ contains two points. Then there exist distinct $x_{1},x_{2}\in B_{i}$ and $y_{1},y_{2}\in B_{j}$ such that $y_{s}=x_{s}+\tau$ for s=1,2. Therefore, $x_{1}-x_{2}=y_{1}-y_{2}$, and the same nonzero difference occurs in both $\Delta B_{i}$ and $\Delta B_{j}$. The converse follows by reversing this argument. □

Suppose the distinct weights are $W=\{w_{1},\ldots ,w_{s}\}$ and exactly $n_{j}$ blocks have weight $w_{j}$. Set

(8) $$M=\sum_{j=1}^{s}n_{j},\;r_{j}=\frac{n_{j}}{M},\;R=\left(r_{1},\ldots ,r_{s}\right).$$

The family is denoted by an (N,W,1,R) VW-OOC. Because a weight $w_{j}$ block contributes $w_{j}\left(w_{j}-1\right)$ ordered differences, an index-one packing satisfies

(9) $$U:=\sum_{j=1}^{s}n_{j}w_{j}\left(w_{j}-1\right)\leq N-1,\;\varepsilon =N-1-U.$$

For maximum correlation one, the refined upper bound of Wu, Zhao, Fan, and Shinohara [6] is

(10) $$M\leq \min_{1\leq j\leq s}\left\lfloor \frac{1}{r_{j}}\left\lfloor r_{j}\left\lfloor \frac{N-1}{\sum_{h=1}^{s}r_{h}w_{h}\left(w_{h}-1\right)}\right\rfloor \right\rfloor \right\rfloor .$$

A VW-OOC meeting (10) is optimal for its prescribed weight profile.

The next proposition is an elementary sufficient test derived here from the existing bound (10). It is not a new upper bound. We retain the designation “Proposition” because this arithmetic observation supports the construction theorems.

**Proposition** **2** (Optimality from small difference deficiency)**.** *Let an (N,W,1,R) VW-OOC have family size M, weight counts $n_{1},\ldots ,n_{s}$, used-difference count U, and difference deficiency ε=N−1−U. Define*

(11) $$t=\left\lfloor \frac{M\varepsilon }{U}\right\rfloor .$$

*If $n_{j}t<M$ for every j, then the code is optimal with respect to *(10)*. In particular,*

(12) Mε<U

*is sufficient for optimality.*

**Proof.**  Since $r_{j}=n_{j}/M$ and $\sum_{h}r_{h}w_{h}\left(w_{h}-1\right)=U/M$, the innermost floor in (10) is

(13) $$\left\lfloor \frac{N-1}{\sum_{h}r_{h}w_{h}\left(w_{h}-1\right)}\right\rfloor =\left\lfloor \frac{M(N-1)}{U}\right\rfloor =M+t.$$

The middle floor in (10) for class *j* is

(14) $$\left\lfloor r_{j}\left(M+t\right)\right\rfloor =n_{j}+\left\lfloor \frac{n_{j}t}{M}\right\rfloor .$$

The assumed inequality makes this value equal to $n_{j}$, and the outer floor in (10) is then $\lfloor n_{j}/r_{j}\rfloor =M$. Thus, every term in the minimum equals *M*. Condition (12) gives t=0 and is therefore sufficient. □

**Remark** **1** (A sufficient condition, not a characterization)**.** *Condition *(12)* is not necessary for profile-optimality. On $Z_{29}$, the blocks {0,1,3} and {0,4,9,15} have disjoint ordered difference sets of sizes *6* and *12*. They give M=2, U=18, ε=10, and R=(1/2,1/2), so Mε=20≥U. Nevertheless, t=1 and $n_{j}t=1<M$ for both classes; the nested bound *(10)* equals *2*. Thus, this code is profile-optimal. More generally, floors and the prescribed profile can certify optimality even when the simpler inequality fails.*

Let *X* be a *k*-element set. A family P of subsets of *X* of size at least two is a *pair packing* if every two-element subset of *X* occurs in at most one block. Define the number of uncovered pairs by

(15) ℓ(P)=k2−∑P∈P|P|2.

A pair packing with ℓ(P)=0 is a pairwise balanced design, denoted by PBD(k,K,1) when its block sizes belong to *K*.

Throughout, {d,e} denotes an unordered pair of distinct base points. It gives two ordered pairs, (e,d) and (d,e), and, hence, two oriented differences e−d and d−e, counted with multiplicity. Even if these residues coincide in the first coordinate, the two orientations remain separate in the product-group difference multiset. Accordingly, each base pair contributes 2L graph differences, and a block of size *w* contributes w(w−1) ordered differences. For a group *H*, we write $H^{*}=H\setminus \left\{0\right\}$; in particular, $Z_{k}^{*}$ means all nonzero residues, not only the units.

### 2.2. CRT Graph Construction and Exact Difference Coverage

Let *p* be a prime and assume

(16) p−1=kL

for positive integers *k* and *L*. Let α be a primitive element of $F_{p}$, and work in the additive group

(17) $$G=Z_{k}\times F_{p}.$$

Because gcd(k,p)=1, the CRT identifies *G* with $Z_{kp}$.

Let $P=\{P_{1},\ldots ,P_{m}\}$ be a pair packing on $Z_{k}$. For 1≤a≤m and 0≤i<L, define

(18) $$S_{a,i}=\left\{\left(d,\alpha^{dL+i}\right):d\in P_{a}\right\}\subseteq G,$$

and put

(19) $$G\left(P\right)=\left\{S_{a,i}:1\leq a\leq m,\;0\leq i<L\right\}.$$

This is the graph coordinate form of Construction 1 and Theorem 2 in [13], with the correlation proof in Section 3.2 of that paper. We use the existing construction and do not claim the graph map itself as new.

Partition the nonzero elements of *G* into

(20) $$\begin{array}{cc} H_{k}^{*} & =(Z_{k}\setminus \left\{0\right\})\times \left\{0\right\}; \end{array}$$

(21) $$\begin{array}{cc} H_{p}^{*} & =\left\{0\right\}\times F_{p}^{*}; \end{array}$$

(22) $$\begin{array}{cc} G_{\mathrm{mix}} & =\left(Z_{k}\setminus \left\{0\right\}\right)\times F_{p}^{*}. \end{array}$$

The first two sets are the punctured coordinate axes, and $G_{\mathrm{mix}}$ is the mixed-coordinate region.

**Lemma** **1** (Unique recovery of the ordered base pair)**.** *For fixed $\delta \in Z_{k}\setminus \left\{0\right\}$, the map*

$$\left(d,i\right)⟼\alpha^{dL+i}\left(\alpha^{\delta L}-1\right),\;0\leq d<k,\;0\leq i<L,$$

*is a bijection onto $F_{p}^{*}$. Thus, a prescribed $\left(\delta ,\eta \right)\in G_{\mathrm{mix}}$ determines exactly one i and one ordered base pair (d+δ,d), with addition in $Z_{k}$.*

**Proof.**  The factor $\alpha^{\delta L}-1$ is nonzero. Multiplication by this factor is a bijection on $F_{p}^{*}$, as is $s\mapsto \alpha^{s}$ for 0≤s<p−1. Euclidean division by *L* writes each such *s* uniquely as s=dL+i in the indicated ranges. Consequently, another graph block producing the same mixed difference must have the same *d*, *i*, and d+δ. The pair-packing property then forces the same base block. □

The following theorem refines the correlation guarantee of [13] to an exact pointwise multiplicity statement.

**Theorem** **1.** *Fix $\left(\delta ,\eta \right)\in G_{\mathrm{mix}}$. Let*

(23) $$s={\mathrm{ind}}_{\alpha }\;\left(\eta {\left(\alpha^{\delta L}-1\right)}^{-1}\right)\in \left\{0,1,\ldots ,p-2\right\},$$

*where ${\mathrm{ind}}_{\alpha }\left(\alpha^{s}\right)=s$. Write uniquely*

(24) s=dL+i,0≤d<k,0≤i<L.

*Then, (δ,η) occurs in ΔG(P) if and only if the pair*

(25) $$\left\{d,d+\delta \right\}\subseteq Z_{k}$$

*occurs in a block of P. When it occurs, its multiplicity is exactly one. No difference generated by a graph block lies in $H_{k}^{*}\cup H_{p}^{*}$.*

**Proof.**  Choose distinct $d,e\in P_{a}$ and put $\delta =e-d\in Z_{k}\setminus \left\{0\right\}$. Their ordered difference in $S_{a,i}$ is

(26) $$\begin{array}{cc} \; & \left(e,\alpha^{eL+i}\right)-\left(d,\alpha^{dL+i}\right)\\ \; & \;=\left(\delta ,\alpha^{dL+i}\left(\alpha^{\delta L}-1\right)\right). \end{array}$$

The factor $\alpha^{\delta L}-1$ is nonzero because 0<δ<k and α has order kL. Thus, a prescribed mixed difference forces

(27) $$\alpha^{dL+i}=\eta {\left(\alpha^{\delta L}-1\right)}^{-1}.$$

Lemma 1 shows that the discrete index and (24) recover exactly one pair (d,i), including when d+δ wraps modulo *k*, since $\alpha^{kL}=1$. The difference occurs precisely when *d* and d+δ lie together in a base block. Since P is a pair packing, that block is unique.

A graph block has distinct first coordinates, so it cannot produce a nonzero difference with first coordinate zero. Its second coordinates are also distinct: if $\alpha^{dL+i}=\alpha^{eL+i}$, then kL divides (d−e)L, so *k* divides d−e, contrary to d≠e in $Z_{k}$. Therefore, no graph difference lies on either coordinate axis. □

The following exact deficiency formula is a consequence of the spectrum theorem; the inherited correlation-one property alone does not specify this leave.

**Corollary** **1.** *The family G(P) is an index-one difference packing in G and hence gives a length-kp VW-OOC with maximum correlation one. Its weight multiset consists of L copies of each base-block size. Moreover,*

(28) $$\varepsilon \left(G(P)\right)=p+k-2+2L\ell \left(P\right).$$

*If P is a PBD, every element of $G_{\mathrm{mix}}$ occurs exactly once and the uncovered nonzero differences are precisely*

(29) $$H_{k}^{*}\cup H_{p}^{*}.$$

**Proof.**  The multiplicity assertion follows from Theorem 1. There are k2−ℓ(P) covered unordered pairs. Each contributes both orientations, and each orientation contributes one difference for every i∈{0,…,L−1}. Thus, the graph family uses

(30) 2Lk2−ℓ(P)=(k−1)(p−1)−2Lℓ(P)

differences in $G_{\mathrm{mix}}$. Since $|G_{\mathrm{mix}}|=\left(k-1\right)\left(p-1\right)$ and neither axis is used, (28) follows. When ℓ(P)=0, the entire mixed-coordinate region is covered. □

Within this construction, using every index 0≤i<L, complete mixed-coordinate coverage holds if and only if ℓ(P)=0. Thus, the PBD condition is necessary as well as sufficient among pair packings. Each missing pair leaves exactly 2L mixed differences uncovered; no axis packing can supply them. A weaker sufficient condition would require a different construction, such as additional blocks with mixed differences.

### 2.3. Completion on the Coordinate Axes

Let A be an index-one cyclic difference packing on the additive group $F_{p}$, and let B be one on $Z_{k}$. Embed their blocks into *G* by

(31) $$\begin{array}{cc} \hat{A} & =\left\{\{0\}\times A:A\in A\right\}; \end{array}$$

(32) $$\begin{array}{cc} \hat{B} & =\left\{B\times \{0\}:B\in B\right\}. \end{array}$$

Table 1 distinguishes the base blocks, generated blocks, axis packings, and their difference multisets. The next theorem is a completion statement for this specific product-group spectrum; general filling methods already occur in [7,8,9].

**Theorem** **2.** *Let P be a pair packing on $Z_{k}$. Then,*

(33) $$V=G\left(P\right)\cup \hat{A}\cup \hat{B}$$

*is an index-one difference packing in G. Under the CRT isomorphism, it is a length-kp VW-OOC with maximum correlation one.*

*If P is a PBD, then*

(34) $$\begin{array}{cc} G^{*}\setminus D\left(V\right)= & \left\{0\right\}\times \left(F_{p}^{*}\setminus D\left(A\right)\right)\\ \; & \cup \left(Z_{k}^{*}\setminus D\left(B\right)\right)\times \left\{0\right\}, \end{array}$$

*where $G^{*}=G\setminus \left\{\left(0,0\right)\right\}$. Consequently,*

(35) ε(V)=ε(A)+ε(B).

**Proof.**  By Theorem 1, all differences from G(P) lie in $G_{\mathrm{mix}}$ and have multiplicity at most one. Differences from $\hat{A}$ lie in $H_{p}^{*}$, while those from $\hat{B}$ lie in $H_{k}^{*}$. These three regions are pairwise disjoint. Each embedded family retains the multiplicities of its original packing, proving the first assertion. If P is a PBD, Corollary 1 shows that the mixed-coordinate region is completely covered, so only the uncovered portions of the two axes remain. □

For an arbitrary pair packing, the same argument gives the more general identity

(36) ε(V)=2Lℓ(P)+ε(A)+ε(B).

**Figure 1:**
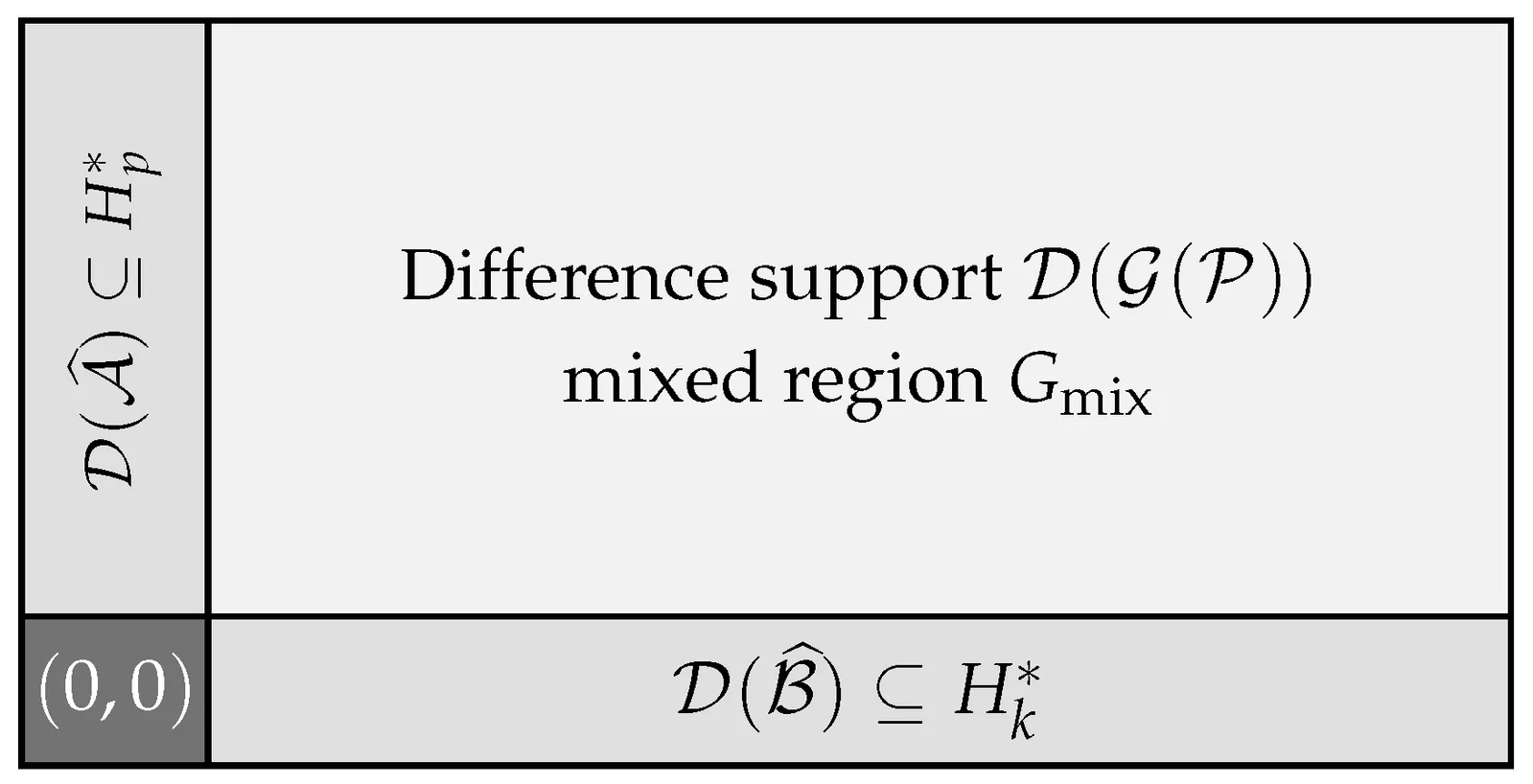
Partition of the product-group difference space. A PBD-generated graph family covers the mixed-coordinate region and leaves both axes unused; that is why the two axes can be completed independently. The labels refer to difference supports, not to block families.

The PBD specialization removes exactly the mixed-coordinate term. Figure 1 illustrates this decomposition.

## 3. Profile-Optimal VW-OOC Families with Weights 3 and 4

### 3.1. Finite-Geometric Seeds and Axis Packings

Finite affine and projective planes provide PBDs with two adjacent block sizes. The following standard deletion construction fixes the exact block counts needed below; see also the finite-field PBD constructions in [14].

**Proposition** **3.** *Let q≥3 be a prime power. The following PBDs exist.*

*1.*  *Deleting one point from PG(2,q) gives*
(37) $$\mathrm{PBD}\left(q^{2}+q,\left\{q,q+1\right\},1\right)$$
  *with q+1 blocks of size q and $q^{2}$ blocks of size q+1.*
*2.*  *Deleting one point from AG(2,q) gives*
(38) $$\mathrm{PBD}\left(q^{2}-1,\left\{q-1,q\right\},1\right)$$
  *with q+1 blocks of size q−1 and $q^{2}-1$ blocks of size q.*

**Proof.**  A projective plane of order *q* has $q^{2}+q+1$ points and the same number of lines; each line has q+1 points, and exactly q+1 lines pass through a point. After one point is deleted, those q+1 lines have size *q*, while the remaining $q^{2}$ lines retain size q+1. An affine plane has $q^{2}$ points and q(q+1) lines of size *q*, with q+1 lines through each point. Deleting one point shortens those q+1 lines and leaves the other $q^{2}-1$ lines unchanged. In both cases, every pair of remaining points is still contained in exactly one block. □

If p−1=kL, Proposition 3 and the graph construction give the following counts. From PG(2,q) with k=q(q+1), one obtains

(39) $$\frac{p-1}{q}\;\mathrm{blocks}\;\mathrm{of}\;\mathrm{size}\;q,\;\frac{q(p-1)}{q+1}\;\mathrm{blocks}\;\mathrm{of}\;\mathrm{size}\;q+1.$$

From AG(2,q) with $k=q^{2}-1$, one obtains

(40) $$\frac{p-1}{q-1}\;\mathrm{blocks}\;\mathrm{of}\;\mathrm{size}\;q-1,\;p-1\;\mathrm{blocks}\;\mathrm{of}\;\mathrm{size}\;q.$$

In each case, all mixed-coordinate differences are used exactly once.

For PG(2,3), deleting one of the 13 points shortens the four incident lines from size 4 to size 3 and leaves nine lines of size 4, on 12 remaining points. For AG(2,4), deleting one of the 16 points shortens five of the 20 lines from size 4 to size 3 and leaves fifteen lines of size 4, on 15 remaining points. Thus, the required seeds have counts (4,9) and (5,15), respectively, and both have exactly the block sizes {3,4}. These are specializations of the standard deletion construction, not new finite geometries. To complete the *p*-axis, we use a cyclic Steiner triple system. A cyclic Steiner triple system of order *v* exists for every v≡1 or 3(mod6), except v=9 [15]. For a prime p≡1(mod6), a set of orbit representatives gives a family $T_{p}$ of (p−1)/6 triples in $Z_{p}$ satisfying

(41) $$D\left(T_{p}\right)=Z_{p}^{*}.$$

To see the difference family property, note that every triple orbit under translation on $Z_{p}$ has size *p*: a nonzero stabilizing translation generates $Z_{p}$, which cannot stabilize a 3-subset when p>3. The p(p−1)/6 triples of the Steiner system therefore form (p−1)/6 orbits. For any a≠0, the pair {0,a} occurs in exactly one translated triple. Equivalently, exactly one representative contains an ordered pair with difference *a*; two such occurrences would translate to two triples containing {0,a}. Hence, the representative differences cover every nonzero residue exactly once.

For the other axis, define

(42) $$B_{12}=\left\{\{0,1,3\}\right\}\;\mathrm{in}\;Z_{12},\;B_{15}=\left\{\{0,1,3,7\}\right\}\;\mathrm{in}\;Z_{15}.$$

The first block uses the six differences {±1,±2,±3}, while the second uses every nonzero residue of $Z_{15}$ except 5 and 10.

### 3.2. Two Infinite Classes and Their Optimality

The next elementary divisibility observation is included for completeness and is not claimed as an independent new result. It gives a lower bound on deficiency for weights 3 and 4.

**Proposition** **4.** *For any index-one difference packing on $Z_{N}$ whose block sizes belong to {3,4},*

(43) ε≡N−1(mod6).

*Consequently, the difference deficiency is at least 5 when N=12p, and at least 2 when N=15p with p≡1(mod6).*

**Proof.**  Every block of size 3 uses 6 ordered differences and every block of size 4 uses 12. Hence, the total number *U* of used differences is divisible by 6, and ε=N−1−U gives (43). Since 12p−1≡5(mod6) and 15p−1≡2(mod6) for p≡1(mod6), the stated lower bounds follow. □

**Theorem** **3.** *The following VW-OOCs are optimal for their explicitly prescribed profiles. The assertions include exact block counts and minimum difference deficiency; they do not assert unrestricted maximum cardinality or novelty of the lengths. The second parameter tuple also has the prior packing completion described in Section 3.3.*

*1.*  *For every prime p≡1(mod12), there exists a profile-optimal*
(44) $$\left(12p,\left\{3,4\right\},1,\left(\frac{2(p+1)}{5p-1},\frac{3(p-1)}{5p-1}\right)\right)\text{-}\mathrm{VW}\text{-}\mathrm{OOC}$$
  *with*
(45) $$n_{3}=\frac{p+1}{2},\;n_{4}=\frac{3(p-1)}{4},\;M=\frac{5p-1}{4}.$$
  *It has difference deficiency *5* and uses a proportion*
(46) $$\rho_{12}\left(p\right)=\frac{12p-6}{12p-1}=1-\frac{5}{12p-1}$$
  *of all nonzero differences.*
*2.*  *For every prime p≡1(mod30), there exists a profile-optimal*
(47) $$\left(15p,\left\{3,4\right\},1,\left(\frac{p-1}{3p-1},\frac{2p}{3p-1}\right)\right)\text{-}\mathrm{VW}\text{-}\mathrm{OOC}$$
  *with*
(48) $$n_{3}=\frac{p-1}{2},\;n_{4}=p,\;M=\frac{3p-1}{2}.$$
  *It has difference deficiency *2* and uses a proportion*
(49) $$\rho_{15}\left(p\right)=\frac{15p-3}{15p-1}=1-\frac{2}{15p-1}$$
  *of all nonzero differences.*

*Both classes are optimal for their prescribed weight profiles and attain the minimum difference deficiency possible for block sizes *3* and *4* at their respective lengths.*

**Proof.**  Table 2 records the graph and two axis contributions separately. For part 1, set k=12 and L=(p−1)/12. Delete one point from PG(2,3) to obtain a PBD(12,{3,4},1) with four triples and nine quadruples. Equations (39) and (41), together with the embedded block $B_{12}$, give

(50) $$\begin{array}{cc} n_{3} & =\frac{p-1}{3}+\frac{p-1}{6}+1=\frac{p+1}{2}, \end{array}$$

(51) $$\begin{array}{cc} n_{4} & =\frac{3(p-1)}{4}. \end{array}$$

The coordinate-axis completion theorem gives maximum correlation one. The *p*-axis and the mixed-coordinate region are completely covered, while $B_{12}$ leaves five nonzero differences unused on the 12-axis. Thus,

(52) $$U=6n_{3}+12n_{4}=12p-6,\;\varepsilon =5.$$

Since 5M<U, Proposition 2 proves profile-constrained optimality. Proposition 4 shows that ε=5 is the smallest possible value for any index-one {3,4} packing of length 12p.

For part 2, set k=15 and L=(p−1)/15. Delete one point from AG(2,4) to obtain a PBD(15,{3,4},1) with five triples and fifteen quadruples. Equations (40) and (41), together with the embedded block $B_{15}$, give

(53) $$n_{3}=\frac{p-1}{3}+\frac{p-1}{6}=\frac{p-1}{2},\;n_{4}=\left(p-1\right)+1=p.$$

The only unused nonzero differences are (5,0) and (10,0) in $Z_{15}\times F_{p}$, so

(54) $$U=6n_{3}+12n_{4}=15p-3,\;\varepsilon =2.$$

Since 2M<U, Proposition 2 proves profile-constrained optimality, and Proposition 4 proves deficiency minimality. □

**Remark** **2.** *Dirichlet’s theorem on primes in arithmetic progressions gives infinitely many primes congruent to one modulo *12* and modulo *30* [16]. Hence, both parts of Theorem 3 define infinite classes.*

**Remark** **3.** *The word “optimal” in Theorem 3 is relative to the exact prescribed profile R. It does not assert maximum cardinality among all index-one {3,4}-VW-OOCs of the same length when the profile is allowed to vary. The profile-independent assertion proved here is instead that the difference deficiency is minimum among all index-one packings with block sizes* 3 *and* 4 *at the stated lengths.*

### 3.3. Explicit Examples and Scope

**Verification procedure.** The two examples are verified by a deterministic procedure using exact integer arithmetic. The procedure checks the PBD pair multiplicities, primitive-element orders, and each axis packing; it then applies the CRT convention t≡d(modk), t≡y(modp) to every product point (d,y). For every block and every ordered pair of distinct support points, it records x−y(modN) and increments an *N*-entry multiplicity array. Checking that every nonzero entry is at most one verifies the entire internal difference multiset, and the zero entries give the exact deficiency. This part costs $O(Mw_{\max}^{2}+N)$, where $w_{\max}$ is the maximum block size.

Independently, the procedure evaluates $|\left(B_{i}+\tau \right)\cap B_{j}|$ by direct set membership for every shift and every i≤j. It excludes τ=0 when taking the autocorrelation maximum, but includes it for distinct blocks. The cases i>j follow by reversing the shift. This independent check costs $O(NM^{2}w_{\max})$. Thus, Table 3 reports exact enumeration, not sampling or a floating-point simulation.

**Length 156.** Take p=13, k=12, L=1, and primitive element α=2. A cyclic Steiner triple system on $Z_{13}$ has base blocks

(55) {0,1,4},{0,2,7}.

Together with the punctured PG(2,3) design and the 12-axis block {0,1,3}, this gives

(56) $$\left(N,n_{3},n_{4},M\right)=\left(156,7,9,16\right),\;R=\left(\frac{7}{16},\frac{9}{16}\right).$$

The family uses 150 of the 155 nonzero differences. Under the CRT labeling specified above, the five uncovered differences are

(57) $$\left\{52,65,78,91,104\right\}\subseteq Z_{156}.$$

The sixteen support sets are listed in Appendix B.

**Length 465.** Take p=31, k=15, L=2, and primitive element α=3. One choice of cyclic Steiner triple-system base blocks on $Z_{31}$ is

(58) $$\begin{array}{cc} \; & \{0,1,3\},\;\{0,4,11\},\;\{0,5,17\},\\ \; & \{0,8,18\},\;\{0,6,15\}. \end{array}$$

The completed code has

(59) $$\left(N,n_{3},n_{4},M\right)=\left(465,15,31,46\right),\;R=\left(\frac{15}{46},\frac{31}{46}\right).$$

It uses 462 of the 464 nonzero differences. The two uncovered differences are

(60) $$\left\{155,310\right\}=\left\{\pm 155\right\}\subseteq Z_{465},$$

which are the CRT images of (5,0) and (10,0).

The recent construction in [13] uses the CRT graph construction to prove maximum correlation one for selected base subsets. The present framework retains that map but changes the central invariant from a correlation upper bound to the complete support of the difference multiset. This reveals that the graph family occupies only the mixed-coordinate region, quantifies the exact effect of uncovered base pairs, and makes the remaining construction problem one-dimensional on each coordinate axis. Table 4 summarizes these structural differences.

Codes with the same lengths, weight set, and correlation parameter occur in the prior literature under different profiles. For the primes in Theorem 3, 12p≡12(mod24), so the fixed-profile family of Jiang, Wu, and Lee [10] applies at length 12p with profile (2/3,1/3). Their fixed-profile results also cover the present length-15p case under profile (1/3,2/3). Related general and perfect-difference-family mechanisms are developed in [8,11]. Table 5 separates these same-length results from the exact profiles constructed here.

At the mechanism level, Jiang, Wu, and Lee use skew starters to obtain regular packings with a prescribed profile and a subgroup leave. Our graph map instead assigns every mixed difference to a recovered base pair, separates two unused axes, and completes them independently. In particular, Lemma 8 in [10], with q=p, leaves $\left\{0\right\}\times (Z_{15}\setminus \left\{0\right\})$ in $Z_{p}\times Z_{15}$. Adding {0}×{0,1,3,7} gives exactly the counts and deficiency of Theorem 3 (2). This is a direct consequence of their regular packing, so the present second family is an alternative uniform realization. Filling the 12-subgroup leave described in Lemma 26 in [10] with one triple gives $\left(n_{3},n_{4}\right)=\left(p,\left(p-1\right)/2\right)$, different from part 1. This distinction does not establish impossibility under arbitrary recursion or block trades.

The product-group constructions of Qin and Zhao are even closer in form; see Theorem 2.1 in [12]. For a prime q=6st+1, their length-12q and length-15q families have block counts

(61) $$\left((6s-2)t,(3s+1)t\right)\;\mathrm{and}\;\left((3s-2)t,(6s+1)t\right),$$

respectively, with deficiencies 11 and 14. The profiles in these unfilled regular families are distinct from those in Theorem 3. This comparison alone does not exclude obtaining a completed tuple by filling their leaves. Indeed, after setting q=p, equality of the respective weight 3 proportions would require

(62) $$18s^{2}t-12st-3s-1=0\;\mathrm{or}\;15st-3s+2=0,$$

and neither equation has a solution in positive integers s,t. Thus, the completed profiles do not equal the displayed unfilled profiles in [12]; no claim about every subsequent filling operation follows from these equations.

General filling constructions and relative difference families with variable block sizes were already developed in [7,8,9,17]. Accordingly, Theorem 2 should be understood as a product-specific completion result made possible by Theorem 1, rather than as the first general filling principle. The principal structural contribution is the proof that this CRT graph family has an exact difference spectrum whose unused part is localized on the two coordinate axes. The one-block axis completions then produce the exact profile-dependent parameters in Theorem 3 and reduce the deficiencies to the congruence-forced minima 5 and 2. We do not claim that all completed parameter tuples are inaccessible to earlier recursive or filling constructions.

The spectrum decomposition also suggests possible extensions based on punctured finite geometries. Candidate seeds include PG(2,q) and AG(2,q), with weight sets {q,q+1} and {q−1,q}, respectively, provided suitable one-dimensional axis packings are available.

## 4. Conclusions

We determined the complete difference spectrum of a CRT-based graph construction for multi-weight OOCs. A pair packing on $Z_{k}$ controls the mixed-coordinate spectrum exactly: each prescribed mixed difference recovers a unique candidate base pair, and every uncovered base pair accounts for 2L uncovered mixed differences. The graph blocks never use the two coordinate axes. When the base packing is a PBD, the mixed-coordinate region is completely covered, and the axes can be completed independently by ordinary cyclic difference packings.

This decomposition leads to a concise construction strategy: choose a PBD seed, generate the mixed-coordinate graph family, and optimize two lower-dimensional axis packings. A small-deficiency argument establishes optimality for the prescribed weight profile. Punctured PG(2,3) and AG(2,4) yield length-12p and length-15p VW-OOCs with weights 3 and 4. Their difference deficiencies are five and two, respectively, which are the minimum values permitted by congruence. The explicit length-156 and length-465 examples have been verified by exact enumeration. The exact profiles and block counts are recorded together with these simultaneous guarantees. Earlier constructions already provide the same lengths and weight set, and the length-15p completed tuple is also obtainable from an earlier regular packing. The contribution established here is the exact-spectrum theorem and its uniform completion mechanism, illustrated by these two fully verified classes.

The spectrum and completion theorems already allow any base-block sizes; they are not restricted to {3,4}. Likewise, Proposition 3 supplies larger punctured-geometric seeds. Obtaining further profile-optimal, minimum-deficiency cyclic families from these seeds remains conditional on suitable axis packings and the corresponding arithmetic bounds.

For a prime-power field $F_{q}$, the exponent-recovery argument still works in $Z_{k}\times \left(F_{q},+\right)$ when k∣(q−1), because $F_{q}^{*}$ is cyclic and every nonzero factor is invertible. When *q* is not prime, however, $\left(F_{q},+\right)$ is not cyclic, so this product cannot be identified with $Z_{kq}$ by the CRT. It gives a group difference packing rather than the cyclic OOC asserted here. Replacing $F_{p}$ by $Z_{m}$ for composite *m* introduces a different obstacle: nonzero residues need not be units, a primitive element need not parameterize all of them, and $\alpha^{\delta L}-1$ may not be invertible. Suitable exponent maps, injectivity conditions, and second-axis packings would therefore need separate proofs. These non-prime cyclic extensions, as well as general prescribed-profile realization at minimum deficiency, remain future problems.

## Figures and Tables

**Table 1:** Notation for the construction and its difference spectrum.

Symbol	Meaning
$P=\{P_{a}\}$	Pair packing on $Z_{k}$ before graph generation
$S_{a,i},\;G\left(P\right)$	One graph block and the family of all graph blocks in *G*
A,B	Difference packings on $F_{p}$ and $Z_{k}$, respectively
$\hat{A},\;\hat{B}$	The corresponding embedded block families in *G*
V	Completed family $G\left(P\right)\cup \hat{A}\cup \hat{B}$
ΔF,D(F)	Ordered difference multiset of a family F and its support
ℓ(P),ε(F)	Uncovered unordered base pairs and unused nonzero differences

**Table 2:** Independent contributions to the completed weight counts. Here, L=(p−1)/k.

*k*	Component	$n_{3}$	$n_{4}$	Block Count
12	Graph family	4L	9L	13L
12	*p*-axis	(p−1)/6	0	(p−1)/6
12	*k*-axis	1	0	1
12	Total	(p+1)/2	3(p−1)/4	(5p−1)/4
15	Graph family	5L	15L	20L
15	*p*-axis	(p−1)/6	0	(p−1)/6
15	*k*-axis	0	1	1
15	Total	(p−1)/2	*p*	(3p−1)/2

**Table 3:** Exact verification summary for the two smallest examples.

*N*	$n_{3}$	$n_{4}$	*M*	Used Differences	Difference Deficiency	Max. Auto-Corr.	Max. Cross-Corr.
156	7	9	16	150/155	5	1	1
465	15	31	46	462/464	2	1	1

**Table 4:** Structural comparison with the construction in [13].

Feature	Construction in [13]	Present Framework
Base object	Subsets of $Z_{k}$ with pairwise intersections at most one	Pair packings with an explicit uncovered-pair count; PBDs singled out as mixed-region seeds
Difference analysis	Direct proof that all relevant correlations are at most one	Exact multiplicity criterion for every product-group difference
Unused differences	Not characterized	Exact deficiency p+k−2+2Lℓ, separated into axis and mixed-coordinate contributions
Additional construction step	Search for further base subsets or weight profiles	Independent cyclic completion on $F_{p}$ and $Z_{k}$
Optimality	Equality in a profile-dependent bound for selected examples	General small-deficiency criterion, two infinite profile-optimal classes, and minimum possible deficiency

**Table 5:** Comparison with explicit same-length {3,4}-VW-OOC families. The cited rows are the unfilled regular packings; the length-15p completed row can also be obtained by filling the cited packing. Entries count base codewords, and ε counts uncovered nonzero differences.

Source	Length	Profile $R=(r_{3},r_{4})$	$\left(n_{3},n_{4};M\right)$	ε
Jiang–Wu–Lee [10]	12p	$\left(\frac{2}{3},\frac{1}{3}\right)$	$\left(p-1,\frac{p-1}{2};\frac{3(p-1)}{2}\right)$	11
Theorem 3 (1)	12p	$\left(\frac{2(p+1)}{5p-1},\frac{3(p-1)}{5p-1}\right)$	$\left(\frac{p+1}{2},\frac{3(p-1)}{4};\frac{5p-1}{4}\right)$	5
Jiang–Wu–Lee [10]	15p	$\left(\frac{1}{3},\frac{2}{3}\right)$	$\left(\frac{p-1}{2},p-1;\frac{3(p-1)}{2}\right)$	14
Theorem 3 (2)	15p	$\left(\frac{p-1}{3p-1},\frac{2p}{3p-1}\right)$	$\left(\frac{p-1}{2},p;\frac{3p-1}{2}\right)$	2

## Data Availability

The mathematical constructions, PBD seeds, CRT mapping, and verification procedure are described in this article. All computations use exact integer arithmetic and the Python 3.12.14 standard library.

## Appendix A. Explicit PBD Seeds

The following labeled blocks are used in the explicit examples. Any bijection from the geometric point set to $Z_{k}$ gives an isomorphic PBD; these labels are fixed only for reproducibility.

### Appendix A.1. The Punctured *PG(2,3)* Design on $Z_{12}$

(A1) $$\begin{array}{cc} P_{12}= & \{\{0,4,5\},\{1,6,9\},\{2,7,11\},\{3,8,10\},\\ \; & \;\;\{0,1,2,3\},\{0,6,7,8\},\{0,9,10,11\},\\ \; & \;\;\{1,4,7,10\},\{1,5,8,11\},\{2,4,8,9\},\\ \; & \;\;\{2,5,6,10\},\{3,4,6,11\},\{3,5,7,9\}\}. \end{array}$$

There are four triples and nine quadruples, and every pair of points in $Z_{12}$ occurs exactly once.

### Appendix A.2. The Punctured *AG(2,4)* Design on $Z_{15}$

(A2) $$\begin{array}{cc} P_{15}= & \{\{0,1,2\},\{3,7,11\},\{4,9,14\},\{5,10,12\},\{6,8,13\},\\ \; & \;\;\{0,3,10,13\},\{0,4,8,12\},\{0,5,7,14\},\{0,6,9,11\},\\ \; & \;\;\{1,3,8,14\},\{1,4,10,11\},\{1,5,9,13\},\{1,6,7,12\},\\ \; & \;\;\{2,3,9,12\},\{2,4,7,13\},\{2,5,8,11\},\{2,6,10,14\},\\ \; & \;\;\{3,4,5,6\},\{7,8,9,10\},\{11,12,13,14\}\}. \end{array}$$

There are five triples and fifteen quadruples, and every pair of points in $Z_{15}$ occurs exactly once.

## Appendix B. Support Sets for the Length-156 Example

Using α=2, the CRT convention in Section 3.3 produces the following supports in $Z_{156}$:

(A3) $$\begin{array}{cccccc} B_{1} & = & \{16,144,149\}, & B_{2} & = & \{57,90,145\},\\ B_{3} & = & \{59,115,134\}, & B_{4} & = & \{10,99,152\},\\ B_{5} & = & \{99,134,144,145\}, & B_{6} & = & \{90,115,144,152\},\\ B_{7} & = & \{10,57,59,144\}, & B_{8} & = & \{10,16,115,145\},\\ B_{9} & = & \{59,145,149,152\}, & B_{10} & = & \{16,57,134,152\},\\ B_{11} & = & \{10,90,134,149\}, & B_{12} & = & \{16,59,90,99\},\\ B_{13} & = & \{57,99,115,149\}, & B_{14} & = & \{0,108,144\},\\ B_{15} & = & \{0,72,132\}, & B_{16} & = & \{0,13,39\}. \end{array}$$

The first thirteen blocks come from the CRT graph construction, $B_{14}$ and $B_{15}$ are the embedded 13-axis Steiner triples, and $B_{16}$ is the embedded 12-axis block.
